# Dr. Elliott Calvin Smith

**Published:** 1916-02

**Authors:** 


**Dr. Elliott Calvin Smith,** Buffalo 1882, died at his home in Corfu, Jan. 8, having returned from the Deaconess Hospital of Buffalo Dec. 28. He was born at Marilla May 30, 1859, living afterward at Akron, Oakfield and East Pembroke. He had practiced in Corfu since his graduation in medicine 34 years ago. He was a graduate of the Oakfield Seminary and of the University of Michigan. The funeral was held Jan. 11, under Masonic auspices.

